# Supplementary material for: Assessing G4-Binding Ligands In Vitro and in Cellulo Using Dimeric Carbocyanine Dye Displacement Assay
Source: Molecules. 2021 Mar 5;26(5):1400. doi: 10.3390/molecules26051400 (PMC7961521; doi:10.3390/molecules26051400)
Supplement: Supplementary file 1 [file molecules-26-01400-s001.pdf]

molecules

MDPI

Supplementary Information

Assessing G4-Binding Ligands In Vitro and in Cellulo Using Dimeric Carbocyanine Dye Displacement Assay

Nakshi Desai [1], Viraj Shah [1] and Bhaskar Datta [1,2,*]

[1] Department of Biological Engineering, Indian Institute of Technology, Gandhinagar 382355, India; nakshi.desai@iitgn.ac.in (N.D.); viraj.s@iitgn.ac.in (V.S.)
[2] Department of Chemistry, Indian Institute of Technology, Gandhinagar, Gandhinagar 382355, India
* Correspondence: bdatta@iitgn.ac.in; Tel.: +79-2395-2427; Fax: +79-2397-2622

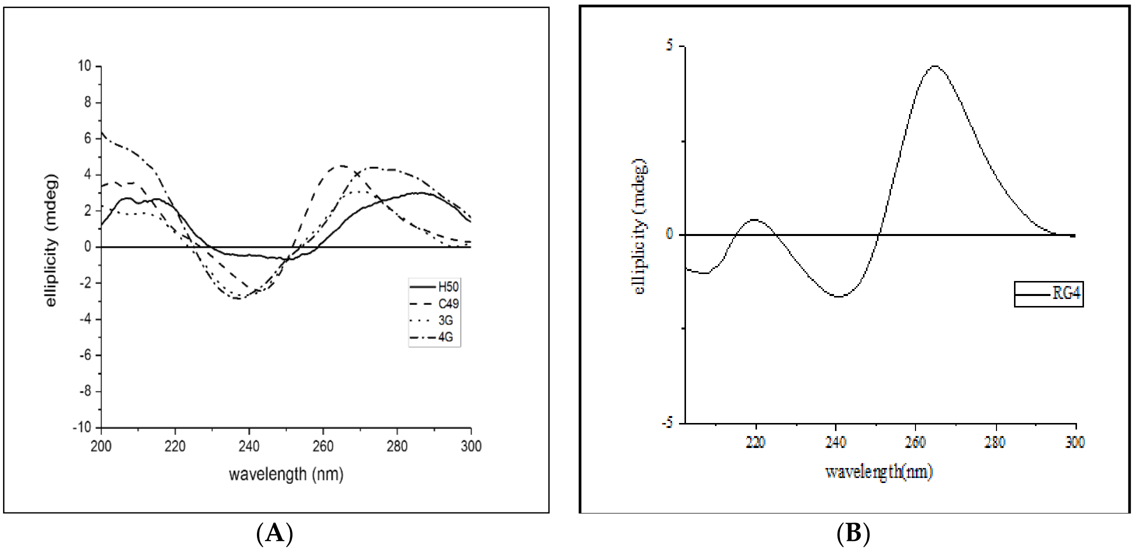

Suplementary Figure S1. (**A**) Overlay of CD Spectra of H50, C49, 3G and 4G DNA oligonucleotide sequences mentioned in Table 1 (**B**) CD Spectra of RG4 RNA oligonucleotide. All spectral scans for CD were performed in presence of 100 mM KCl and 10mM Tris HCl pH 7.2.

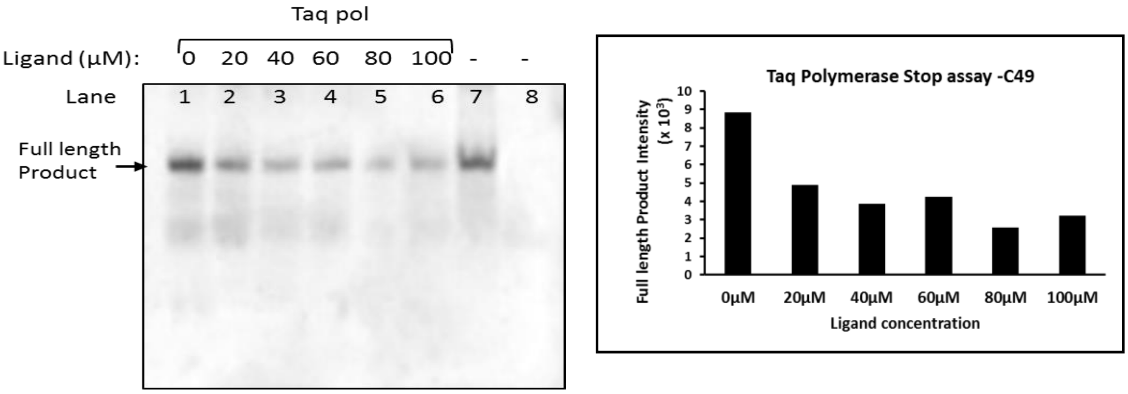

Suplementary Figure S2. *Taq* Polymerase stop assay of C49 DNA sequence analyzed using 15% denaturing PAGE in presence of 0 to 100 µM of ligand B6,5. ImageJ representation of ligand effect shown on the right side.

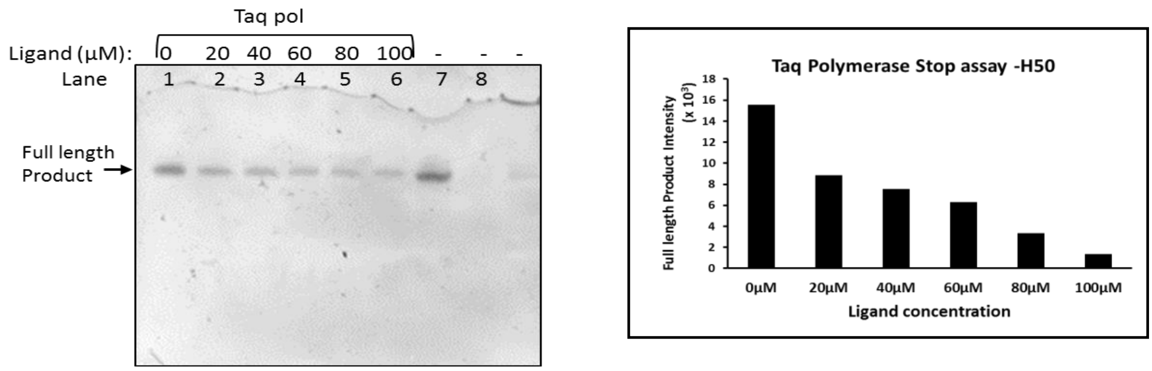

Suplementary Figure S3. *Taq* Polymerase stop assay of H50 DNA sequence analyzed using 15% denaturing PAGE in presence of 0 to 100 µM of ligand B6,5. ImageJ representation of ligand effect shown on the right side.

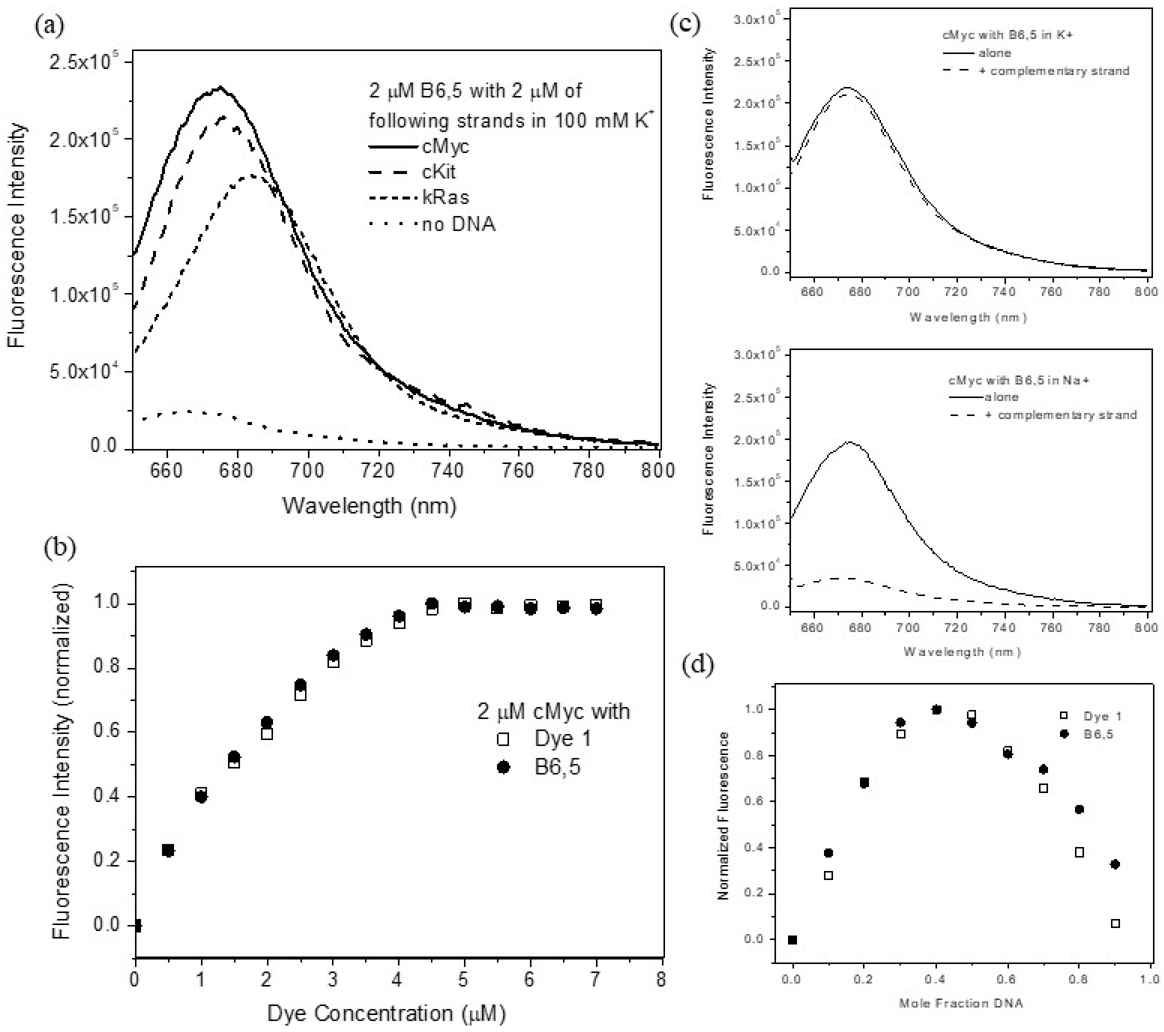

Suplementary Figure S4. (a) Fluorescence spectral response of B6,5 in presence of various physiologically relevant G4 DNA sequences; (b) comparative fluorescence titration of (dimeric carbocyanine dye) Dye 1 and B6,5 on 2 µM c-myc; (c) fluorescence spectral comparison of B6,5 with c-myc alone and in presence of complementary strand, in K^+ and Na^+; (d) Job's plot comparison of Dye 1 and B6,5 with c-myc. All experiments were performed in 10 mM Tris HCl pH 7.2.

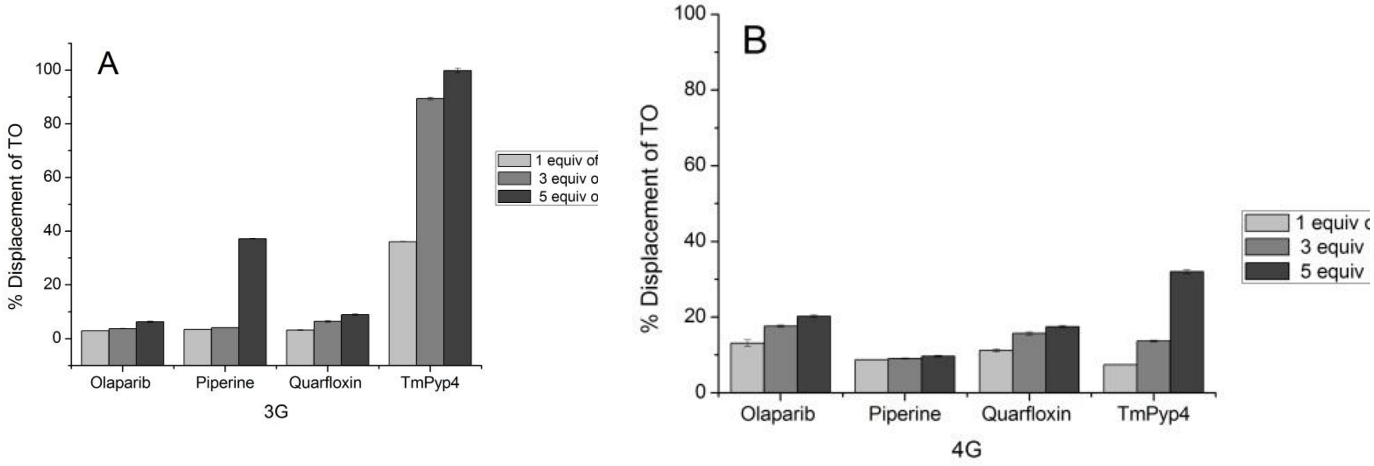

Suplementary Figure S5. Fluorescent dye displacement in (A) 3G and (B) 4G sequences using different G4 specific ligands with Thiazole orange as reporter dye.

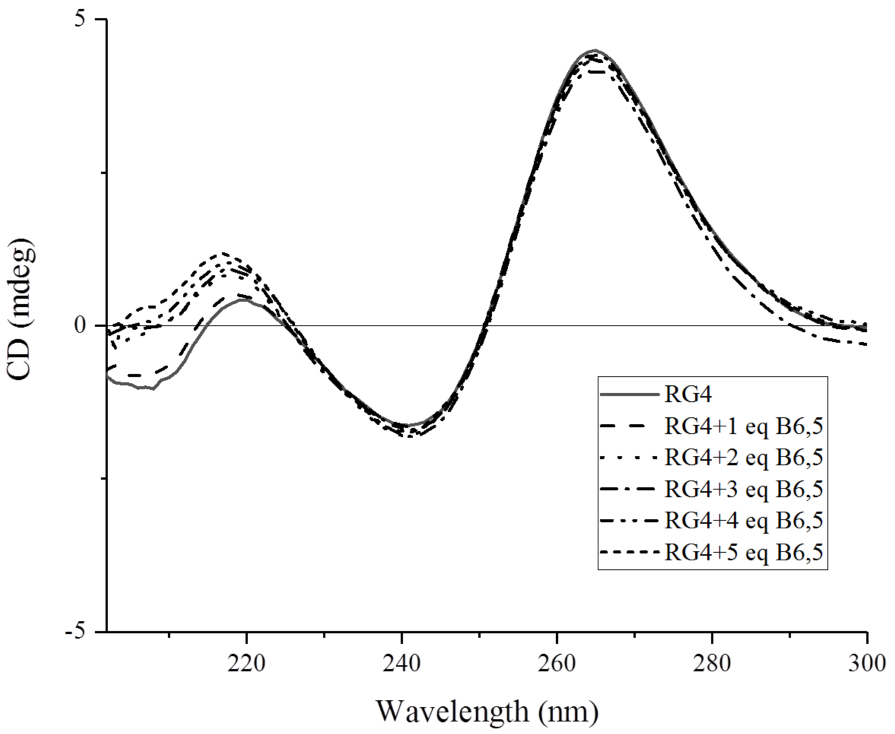

Suplementary Figure S6. CD spectra for titration of ligand B6,5 in increasing molar equivalence from 0 to 5 molar equivalent against template RNA; RG4 (4μM) in 100 mM KCl.

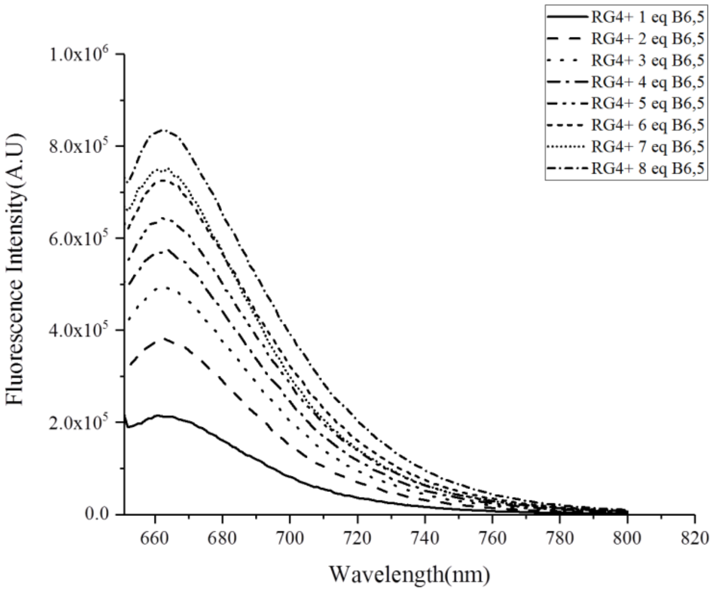

Suplementary Figure S7. Fluorescence Spectroscopy of RG4 and titration with increasing molar equivalence of ligand B6,5 from 0 to 8 equivalence.

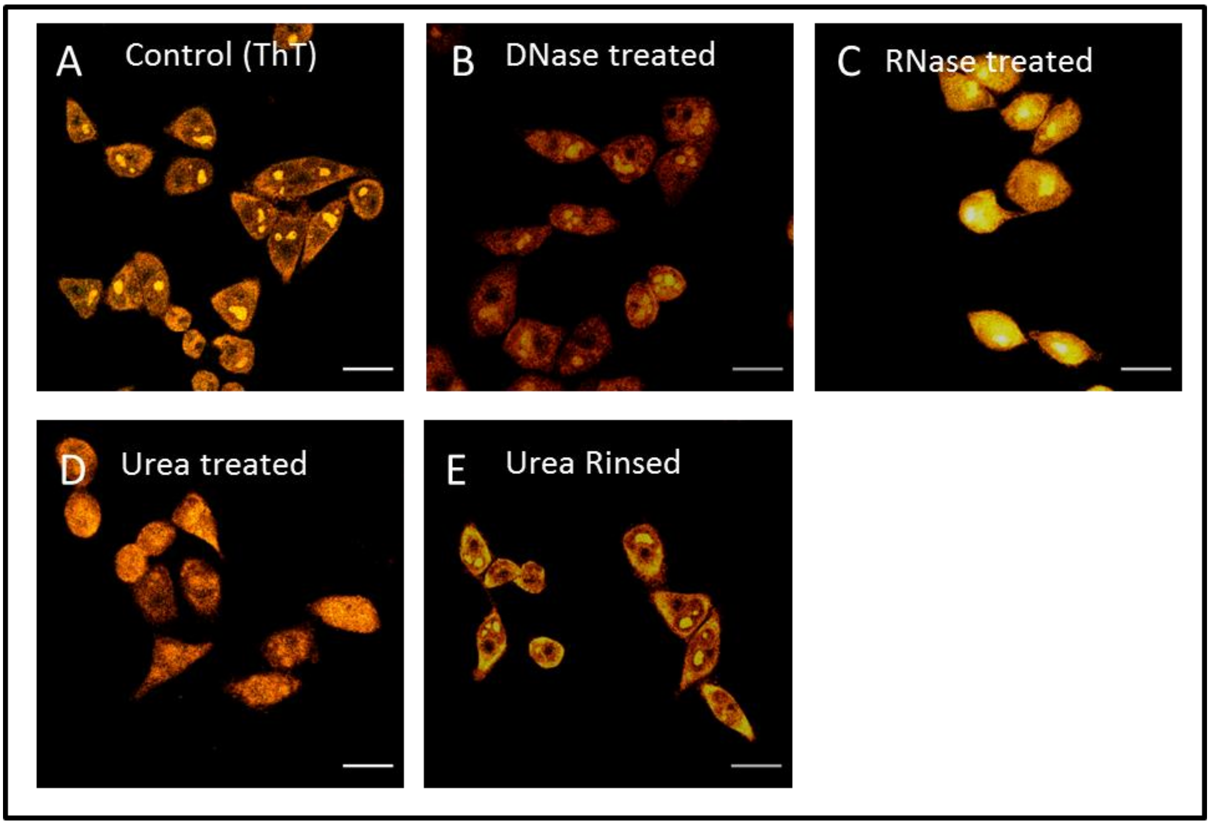

Suplementary Figure S8. CLSM imaging of fixed HeLa cells with (A) ThT (B) DNase (C) RNase (D) Urea treated (E) Urea rinsed with PBS. The nucleus is co-stained using Propidium iodide (PI). Scale bar 50 μm. For clarity images are presented in pseudo colors of green for ThT and red for PI.

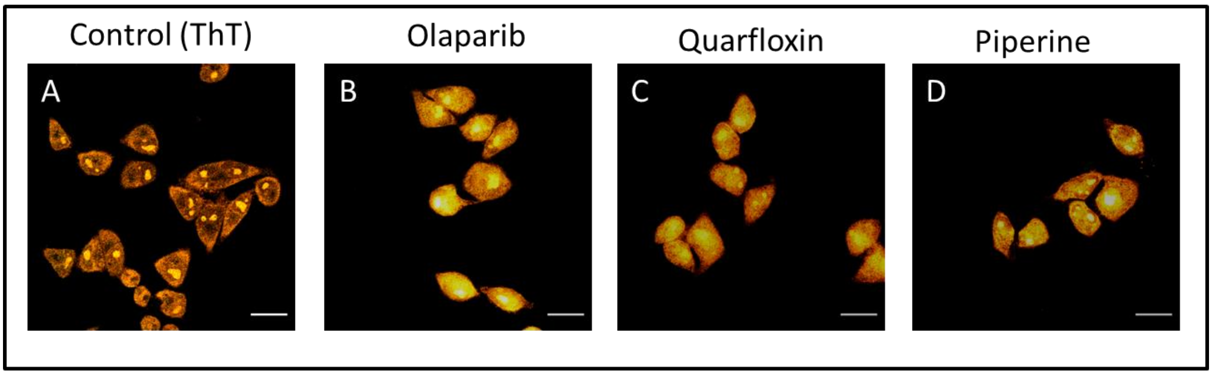

Suplementary Figure S9. CLSM imaging of fixed HeLa cells with (A) ThT (B) Olaparib treated (C) Piperine treated and (D) Quarfloxin treated. The nucleus is co-stained using Propidium iodide (PI). Scale bar 50 μm. For clarity images are presented in pseudo colors of green for ThT and red for PI.
